# Susceptibility and Severity of Viral Infections in Obesity: Lessons from Influenza to COVID-19. Does Leptin Play a Role?

**DOI:** 10.3390/ijms22063183

**Published:** 2021-03-20

**Authors:** Valeria Guglielmi, Luca Colangeli, Monica D’Adamo, Paolo Sbraccia

**Affiliations:** Department of Systems Medicine, University of Rome Tor Vergata, 00133 Rome, Italy; luca.colangeli.ptv@gmail.com (L.C.); dadamo@med.uniroma2.it (M.D.); sbraccia@med.uniroma2.it (P.S.)

**Keywords:** obesity, leptin, leptin resistance, immune system, viral infections, Sars-CoV2, influenza

## Abstract

The recent pandemic Sars-CoV2 infection and studies on previous influenza epidemic have drawn attention to the association between the obesity and infectious diseases susceptibility and worse outcome. Metabolic complications, nutritional aspects, physical inactivity, and a chronic unbalance in the hormonal and adipocytokine microenvironment are major determinants in the severity of viral infections in obesity. By these pleiotropic mechanisms obesity impairs immune surveillance and the higher leptin concentrations produced by adipose tissue and that characterize obesity substantially contribute to such immune response dysregulation. Indeed, leptin not only controls energy balance and body weight, but also plays a regulatory role in the interplay between energy metabolism and immune system. Since leptin receptor is expressed throughout the immune system, leptin may exert effects on cells of both innate and adaptive immune system. Chronic inflammatory states due to metabolic (i.e., obesity) as well as infectious diseases increase leptin concentrations and consequently lead to leptin resistance further fueling inflammation. Multiple factors, including inflammation and ER stress, contribute to leptin resistance. Thus, if leptin is recognized as one of the adipokines responsible for the low grade inflammation found in obesity, on the other hand, impairments of leptin signaling due to leptin resistance appear to blunt the immunologic effects of leptin and possibly contribute to impaired vaccine-induced immune responses. However, many aspects concerning leptin interactions with inflammation and immune system as well as the therapeutical approaches to overcome leptin resistance and reduced vaccine effectiveness in obesity remain a challenge for future research.

## 1. Introduction

Obesity is a worldwide pandemic resulting from a combination of genetic, behavioral, and environmental variables and that dramatically increases morbidity and mortality. Indeed, it has been recognized as a leading cause of major health issues, particularly metabolic, cardiovascular, and oncologic diseases [1,2,3].

The pathophysiological interactions between obesity and viral infectious diseases have recently gained increasing attention after a strong association between obesity and poor outcome in pandemic H1N1 influenza and Sars-CoV2 infections was observed [4,5]. Although the underlying mechanisms are not well established, a number of potential factors may be involved [6].

Obesity complications, respiratory dysfunction, and pharmacological issues have been proposed as possible mechanisms [7]. However, obesity is also associated with impaired immune responses, suggesting a link between metabolic control and immune tolerance [8].

Among the multiple mechanisms by which obesity could impair immunity, the chronic unbalance in the hormonal and adipocytokine microenvironment seems to be a major determinant in the severity of viral infections in obesity.

Herein we review the available evidence on the various aspects of the association between obesity and viral infections and the current understanding of the possible mechanisms underlying this susceptibility to worse outcomes, with particular focus on the role of leptin.

## 2. Obesity and Risk of Infections

Beyond causing non-communicable conditions, chronically positive energy balance and obesity represent an ideal set-up for getting communicable diseases. Indeed, several epidemiological data suggest that body weight is associated with an increased infection risk [9].

However, with regard to viral infections, data in obese population are so far limited. Two metanalysis showed that patients with obesity had a significantly higher risk for influenza infection (OR: 1.29, 95% CI: 1.11–1.49) [10] with a U-shaped relationship between BMI and the risk of influenza-related pneumonia [11]. In The International Severe Acute Respiratory and Emerging Infection Consortium (ISARIC) Clinical Data Report 20 November 2020, among the reported 95966 clinical COVID-19 cases (93.3% laboratory-confirmed cases) from 42 countries across multiple continents, the prevalence of obesity was 13.4% [12]. A pooled data analysis of 20 studies assessing the association between individuals with obesity and the risk of testing positive for COVID-19 showed that the odds of obese individuals being COVID-19 positive were 46% (OR: 1.46; 95% CI: 1.30–1.65) higher than not obese individuals [13]. Obesity was also more prevalent in patients admitted to intensive care unit (ICU) for COVID-19 compared to a control group of patients admitted to ICU for a non SARS-CoV-2-related acute respiratory disease during the previous year, suggesting a greater susceptibility of obese patients to SARS-CoV-2 infection [14].

The evidence is more substantial for bacterial infections, and among these, in particular for nosocomial rather than community-acquired infections. This discrepancy may be imputable to the fact that the BMI is usually recorded in surgical patients, enabling retrospective studies of this association, but not in hospital admissions for other reasons or in case of community-acquired infections [6]. As a matter of fact, obesity is recognized as a strong independent risk factor of postoperative surgical site infection [15,16,17,18,19]. But, more extensively, obesity has been also identified as an independent risk factor for nosocomial pneumonia, Clostridium difficile colitis, bacteremia, and wound infections [6,20,21]. The effect of obesity on infection risk was especially addressed in a registry-based analysis of 37,808 participants from the Danish Blood Donor Study in which obesity resulted to be associated to a 50% increased rate of nosocomial infections. Respiratory tract infections and those of the skin and subcutaneous tissue were more frequent in obese females and males respectively, whereas abscesses [22], urinary tract infections, and pyelonephritis [23] were more frequent in obese irrespective of gender. Increased rates of bacterial or fungal skin infections in obese have been also reported in other studies [24,25,26].

## 3. Obesity and Viral Infection Outcomes, from Influenza A Virus to SARS-COV-2

Recent data suggest that obesity increases not only the risk of getting infections but also infection-related rates of hospitalization, morbidity, and mortality.

One hundred years back, the influenza virus triggered the 1918 “Spanish Flu” pandemic, which caused 50–100 million deaths [27]. However, in those days obesity was not widespread in the society, while rather undernutrition was an issue. Since then, other pandemics caused by zoonotic transfer of the virus from animals to humans have taken place, but none created such an adverse impact as the Spanish Flu in 1918.

Only following the H1N1 pandemic in 2009, obesity was recognized as an independent risk factor for hospitalization and increased mortality due to influenza [4,9]. Indeed, in California 51% of 534 hospitalized adult patients with H1N1 infection were obese, and 61% of the mortality occurred in these individuals [28]. Obese individuals infected with H1N1 virus had a two-fold higher risk to be admitted in ICU [29] and, once admitted to ICU, were at greater risk of developing pneumonitis [30] and consumed more ICU resources compared to non-obese [31]. Obesity was also shown to increase both the length of stay in ICU and the need for mechanical ventilation [32] and, among hospitalized patients, obese and morbidly obese required earlier antiviral therapy for severe influenza H1N1 than non-obese patients [33].

Data analysis from cohort studies indicated that obese individuals were at higher risk of hospitalization and had a longer hospital stay also as a result of seasonal (A/H3N2) influenza infection compared with normal weight individuals [34,35].

Prior to the 21st century, coronaviruses (CoVs) were considered pathogens of great relevance in veterinary medicine but with a reduced impact on human health [36]. However, in the past two decades novel CoVs have emerged and caused global concern for human health with the epidemics of severe acute respiratory syndrome (SARS) in 2002–2003 and Middle East respiratory syndrome (MERS) in 2012 [37,38]. Though these viruses did not spread efficiently from human to human, both SARS and MERS had a high fatality rate of 9.5 and 34.4%, respectively [39]. Invariably, chronic conditions resulted in increased MERS severity [40,41,42,43] and, according to a metanalysis, obesity was present in 16% of the hospitalized patients [44].

Currently, we have been facing a novel CoV (SARS-CoV-2) pandemic since the end of 2019 causing a respiratory disease, called COVID-19. According to COVID-19 surveillance reports of European Centre for Disease Prevention and Control (ECDC) and Centers for Disease Control and Prevention (CDC) from US, about 91.5% of adult patients with severe COVID-19 present at least one underlying medical condition [45] including obesity as well as hypertension, diabetes, cardiovascular disease, chronic respiratory disease, chronic kidney disease, immune compromised status, cancer, and smoking [46]. Thus, also in COVID-19 pandemic, patients with obesity appear at greater risk for increased disease severity and hospitalization [47]. In a data analysis from 5700 SARS-CoV-2 patients admitted to hospitals belonging to Northwell Health of New York between 1 March 2020 and 4 April 2020, hypertension (56.6%), obesity (41.7%), and diabetes (33.8%) were the most common comorbidities [48]. Obesity was even the most commonly associated comorbidity accounting for 48% of the ICU admissions due to SARS-CoV-2 in a Spanish report [49]. Similarly, at a single center in France, 47.6% of patients admitted to ICU had BMI > 30 Kg/m^2^ and 28.2% BMI > 35 Kg/m^2^ [14]. Positive associations between increasing BMI and the risk of hospitalization and adverse outcomes were highlighted in several reports [50]. Of interest, available data suggest that the relationship between BMI and severity of SARS-CoV-2 infection is more relevant in younger people. Indeed, although older age increased the risk for severe COVID-19, in younger patients the most critical form of the infection was more likely associated to obesity [51]. Accordingly, in retrospective analysis of a cohort of 10,862 individuals hospitalized for COVID-19 the association between increased BMI and disease severity stratified by age was stronger for people <59 years old [52].

## 4. Obesity and Viral Shedding

Prolonged viral shedding has been described in people with obesity [53]. Indeed, symptomatic obese adults shed influenza A virus 42% longer, with predicted mean shedding times of 5.23 days versus 3.68 days in lean subjects, potentially causing long-term transmission [54]. In a small cohort of 100 consecutive patients with COVID-19, longer time (19 ± 8 days) to SARS-CoV-2 negativity in nasopharyngeal swabs was reported compared to nonobese patients (13 ± 7 days) [55], although in some other studies marginal or no difference in time of viral load clearance was observed [50].

This could be imputable to the fact that several types of viruses, including adenovirus Ad-36, influenza A virus, HIV, and cytomegalovirus, can utilize the adipose tissue as a reservoir [56], thus benefiting from the obesity status. With regard to SARS-CoV-2, its binding to angiotensin-converting enzyme 2 (ACE2) receptor has been recognized as a critical step for virus entry into cells, so that ACE2-expressing tissues become direct viral targets displaying progressive pathological alterations up to organ failure in most severe cases [57]. According to a search of a public gene expression database, in human subcutaneous and visceral adipose depots ACE2 expression seems even higher than in respiratory epithelium, enabling adipose tissue to serve as a functional reservoir also for SARS-CoV-2, especially in obese individuals [58]. Nevertheless, this hypothesis needs more substantive experimental proofs since ACE2 mRNA transcripts in visceral adipose tissue were not different in obese compared with normal and underweight individuals [59].

The prolonged viral shedding in obesity has been also putatively attributed to the higher ventilation volumes of obese individuals. This is suggested by the observation that the positive association between BMI and influenza virus RNA in aerosols from exhaled breath was significant especially in males [60].

## 5. Obesity and Response to Vaccine and Antivirals

Defective humoral and cellular immune responses to vaccination (for multiple different viruses) have been observed in people with obesity, leaving them more vulnerable to infection [61]. Indeed, a lower CD8+ T-cell response and a more pronounced decline in influenza antibody titers 12 months after vaccination have been reported in people with obesity compared to individuals with normal weight [62]. In response to the objection that assessment of antibody titers alone may provide misleading information on vaccine effectiveness, analysis of peripheral leukocytes from obese patients vaccinated with trivalent influenza vaccine indicated a positive correlation between vaccine response and B-cell function [63]. Suboptimal immune responses to hepatitis B vaccines in people with BMI > 35 Kg/m^2^ have been reported in a small cohort of 68 individuals who showed lower antibody titers and vaccine-specific- CD4+ T-cell response after the third vaccine dose [64]. In contrast, in Pfizer-BioNTech and Moderna mRNA COVID-19 vaccine trials, even if not powered to detect differences between subgroups and limited by incomplete stratification of high-risk groups, efficacy data appear similar in individuals with and without obesity, at least in the median follow-up period of 2 months in the two published reports [65,66].

With regard to antivirals, an important question is how obesity-related pharmacokinetic and pharmacodynamic changes might affect their efficacy. Although reports on neuraminidase inhibitor oseltamivir, an antiviral used to treat influenza virus infection, suggest that dose adjustment for obese patients is not necessary from a pharmacokinetic perspective, whether the dosage is appropriate to control viral replication is so far unknown [32]. However, both obese and lean mice treated with weight-adjusted dosages of oseltamivir before and during pH1N1 infection showed similar rates lung epithelial cell regeneration and were both completely protected from influenza-related mortality [67].

## 6. Mechanisms That Make Obese Patients Vulnerable to Infections

**Figure 1:**
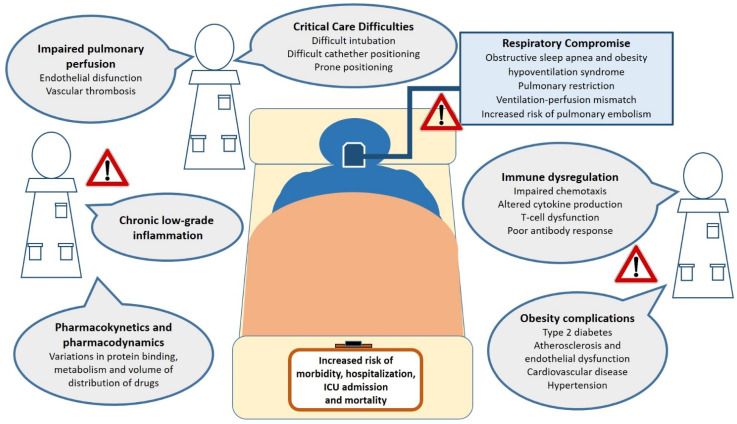
Obesity-related factors associated to adverse clinical outcomes from viral infections in both specialty and intensive care settings. Potential mechanisms by which obesity may augment the risk of critical illness and death from viral infections. These include chronic inflammation, impairment of respiratory function and pulmonary perfusion, critical care management difficulties, immune dysfunction, metabolic and cardiovascular complications of obesity. Abbreviations: ICU, intensive care unit.

As mentioned, obesity implicates a risk of complication, comorbid secondary infections, prolonged hospitalization [68], and the development of acute respiratory distress syndrome [69]. As potential causes of this worse clinical outcome, the dynamic of pulmonary ventilation with reduced diaphragmatic excursions and a relative increase in anatomical death space in obese individuals should be considered [70]. Other mechanisms include decreased pulmonary perfusion, metabolic and vascular complications of obesity, hormonal axys dysregulation, as well as practical considerations when managing obese patients in critical care settings [71] (Figure 1).

In addition, as known, the adipose tissue is dynamically involved in the pathogenesis of obesity complications through a complex network of endocrine, autocrine, and paracrine signals mediated by various adipokines. The obesity-related chronic low-grade inflammation, characterized by raised levels of pro-inflammatory molecules (such as leptin, resistin, chemokine (C-C motif) ligand 2 (CCL2), interleukin (IL)-6, IL-1β, IL-8, tumor necrosis factor (TNF)-α) and decreased levels of adiponectin, may have negative effects on the lung parenchyma and bronchi [72]. This chronic inflammatory milieu, together with T-helper (Th)1 (antiviral action of interferon gamma –IFN-γ-) to Th2 (anti-inflammatory interleukins such as IL-4, -5, -10, and -13) immune response shift induced by viruses to evade host immunity, can be detrimental to the endothelium leading to vascular complications [73].

Moreover, many reports support the notion that a chronic inflammatory status entails a chronic state of immune deregulation, which may interfere with immune homeostasis and the effectiveness of the innate and adaptive immune response [74,75].

With regard to the innate response, when an antigen is presented, as above-mentioned, the obesity-related chronic inflammation hinders further macrophage production of IFN-γ that is known to have direct antiviral actions [76]. In line with this, monocytes isolated from individuals with obesity and diabetes exhibited greater susceptibility to SARS-CoV-2 infection ex vivo [77]. This is due to an upregulation of suppressor of cytokine signaling (SOCS) proteins which are involved in the inhibition of IFN-α/β JAK/STAT signaling. Indeed, obese subjects exhibit increased basal levels of SOCS3 but a significantly lower expression of SOCS3 and SOCS1 in peripheral blood mononuclear cells (PBMCs) after stimulation compared with non-obese subjects, with consequent decreased ability to produce IFN-α and IFN-β in response to Toll-like receptor ligands [78].

The reduced and delayed capacity to produce IFNs in contrast to viral replication could also sustain viral RNA replication and also favor the appearance of novel, more virulent viral strains [79].

Besides macrophages, various effects of obesity on other leukocyte subpopulations including dendritic cells (DCs), natural killer (NK) cells, and neutrophils have been reported but the findings are often contradictory [80,81]. In particular, in some obese human cohorts, circulating DCs were reduced in number and less responsive to ex vivo stimulation with TLR agonists, NK cells were decreased and neutrophils showed increased production of inflammatory free radicals upon stimulation compared to non-obese controls [75,82] but not in others [83,84]. This could have a negative impact on the bridge between non-specific innate immunity and antigen-specific adaptive immunity which in fact relies on appropriate actions of immune cells from both the lymphoid and myeloid lineages.

As a matter of fact, the adaptive (B- and T-cell) responses show reduced efficacy in obesity and obese mice showed a decrease in key transcripts for early lymphoid commitment likely driven by defects in bone marrow environment [85]. Obesity has been associated with increased activation of pro-inflammatory Th1 and Th17 cells and reduction in anti-inflammatory Th2 and regulatory T (Treg) cells [86]. However, the literature on how human obesity modulates B and T cells’ specific response to viruses is limited. After ex vivo stimulation with H1N1 virus, CD8+ and CD4+ T cells produced significantly more IL-5, whereas IFN-γ production trended lower, compared to normal weight subjects [83]. The T-cell response is increasingly considered pivotal in reducing susceptibility to SARS-CoV-2 infection as well as disease severity [87]. With regard to specific T-cell subsets, while in obese donors no difference in the levels of αβ T cells has been reported, γδ T cells exhibit number reduction, a skewed maturation, and a blunted antiviral IFN-γ response to antigen presentation [88] mirroring the immune deficits seen in elderly and thus supporting the “adipaging” hypothesis [89].

As far as B cells are concerned, few studies in humans have directly investigated whether B cell function is impaired in obesity. In human B cells ex vivo, IgM but not IgG levels positively correlated with increasing BMI upon BCR/TLR9 stimulation [90]. Also diet-induce obesity (DIO) mice studies suggest that obesity impairs antibody production [91] as hemagglutination inhibition titers were reduced and completely blunted by 7 and 35 days post influenza infection, respectively [90].

The mechanisms by which obesity impairs immunity are likely pleiotropic. Besides the contribution of obesity complications such as type 2 diabetes, several other factors exert immunomodulatory effects possibly contributing to the increased infection risk. These factors include physical inactivity, nutritional aspects, and a chronic unbalance in the adipocytokine microenvironment.

Reduced physical activity, which is common among obese patients, per se impairs immune response [92]. In fact, exercise not only has a positive impact on energy balance, but also restores leptin response, type I IFN responsiveness, anti-influenza virus-specific IgG2c antibody production, and CD8+ T-cell percentage in bronchoalveolar lavage in obese mice [93].

Fatty acid status could also play a role, since circulating essential long chain n-3 polyunsaturated fatty acids (PUFA), including docosahexaenoic acid (DHA), that are low in obese individuals compared to lean [94], display immunomodulatory properties, namely impairing many aspects of innate and adaptive immunity [95].

Finally, also the higher leptin concentrations, encompassed in the above-mentioned chronic unfavorable hormone milieu observed in obesity, seem to contribute substantially to such dysregulation of the immune response, as detailed next.

## 7. Leptin and Immunometabolism

A link between body weight, adipose tissue, and immunity has been hypothesized for a long time, but the precise molecular mediators were unknown until the discovery of leptin in 1994. Leptin is a non-glycosylated hormone of 146 aminoacids with a tertiary structure resembling that of members of the long-chain helical cytokine family (that includes IL-6, IL-11, IL-12, LIF, G-CSF, CNTF, and oncostatin M) [96]. Functionally, leptin is mainly synthesized in adipose cells in response to food intake and energy balance to provide information to the brain to control feeding and metabolism. Indeed, leptin circulating levels are proportional to the body fat mass and dramatically altered in condition of both chronic negative and positive energy balance so that malnutrition leads to hypoleptinemia, while obesity to hyperleptinemia [97]. Besides its central role in appetite and body weight homeostasis by inducing anorexigenic factors (as cocaine-amphetamine-related transcript), suppressing orexigenic neuropeptides (as neuropeptide Y) in hypothalamus and by influencing energy expenditure, leptin has a regulatory role in multiple important physiologic functions within immune, hematopoietic, neuroendocrine, and reproductive systems, as well as in bone metabolism and inflammation [98].

Leptin has an emerging regulatory role in metabolism-immune system interplay [99], being a cornerstone of the new field of research termed immunometabolism. Indeed, the capacity to store energy finely regulated by the crosstalk between the adipose tissue and the brain and mainly orchestrated by leptin, is entangled with the capacity of this adipokine to activate or extinguish certain physiological responses on the basis of energy availability. One of these responses consists in the activation of immune system to control infections. The connection between metabolism and immune system relies on a complex network of cytokines and neuropeptides that act on peripheral nervous system and endocrine axes, and are ultimately regulated in a central manner [100].

In this context, the central effect of leptin in the hypothalamus is mediated by the activation of the sympathetic nervous system [101] and, to a lesser extent, by the inhibition of the hypothalamic-pituitary-adrenal axis [102].

## 8. Leptin and Immune System

Leptin is also produced by inflammatory regulatory cells and upregulated in response to several inflammatory mediators (i.e., LPS and inflammatory cytokines such as TNF-α, IL-6, and IL-1β), so that serum leptin concentrations highly increase during acute infection, inflammation, and sepsis [103]. Since immune cells express leptin receptor (LepR) on their surface, leptin can perpetuate its effects on these cells through autocrine and/or paracrine signals and contribute to the development of a loop of acute phase reactants [104] and chronic inflammation.

So it is not surprising that leptin, which is almost invariably elevated in obese patients, has been recognized as one of the adipokines mediating the pro-inflammatory state of obesity [105]. In this context, inflammatory cytokines not only stimulate leptin mRNA expression, but also the short-term release of leptin stored in adipose tissue [106].

Not exclusively adipose and immune cells produce leptin in the context of the inflammatory response upon infection. Indeed, human and mice lung epithelial cells in bacterial and viral pneumonia have shown significantly greater leptin staining as compared with uninfected lungs. Concentrations of leptin also increase in bronchoalveolar lavage samples after endotoxin inhalation in both humans and mice [107], in intestinal epithelium upon infection [108] or in animal model of sepsis [109].

**Figure 2:**
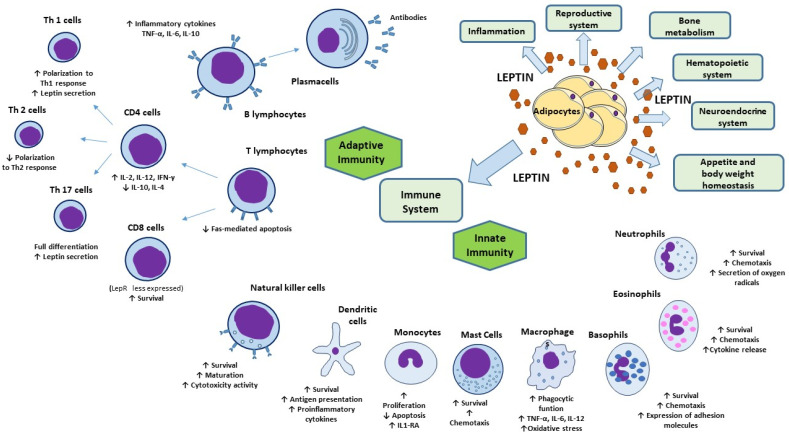
Physiologic functions of leptin and its effects on innate and adaptive immunity. Besides its key role in appetite and body weight homeostasis, leptin exerts physiological functions within immune, hematopoietic, neuroendocrine, and reproductive systems, as well as in bone metabolism and inflammation. In innate immunity, leptin modulates the activity and function of neutrophils, monocytes/macrophages, eosinophils, mast cells, NK cells, and DCs. In adaptive immunity, leptin affects the maturation and survival of T cells. On memory T cells leptin favors the switch toward Th1 cell responses and facilitate Th17 responses and, contrariwise, negatively affects the expansion of Treg. Leptin activates also B-cell responses. Abbreviations: DC, dendritic cells; IL, interleukin; IL1-RA, interleukin 1 receptor-agonist; IFN-γ, interferon-γ; NK, natural killer; Th, T helper; TNF-α, tumor necrosis factor-α; Treg, regulatory T cells.

Besides its role as a mediator of inflammation, leptin has multiple direct and regulatory immune functions (Figure 2). The first evidence of a possible effect of leptin in immune system regulation arises from the observation that LepR belongs to class I cytokine superfamily. In particular, LepR shares signaling capabilities with IL-6-type cytokine receptors relying on JAK/STAT pathway for signal transduction [110]. Alternatively, LepR could activate ERK 1/2, p38 MAPK, JNK, PKC, and PI3K/Akt pathways [111]. Even the wide distribution of LepR which, as said, is expressed on the membrane of both peripheral and bone marrow-derived immune cells, supports the multiple roles of leptin in the immune system [112].

Additionally, conditions of hypoleptinemia have proven that leptin is necessary for the full functionality of immune system. In fact, leptin deficiency increases susceptibility to infections [113] and infection-related mortality [114] and is associated to cytokine dysregulation.

In human monocytes, leptin stimulates proliferation [115], prevents apoptosis [116], upregulates the expression of activation markers, such as CD25, CD38, CD69, CD71 [115] and interferon-gamma-inducible protein-10 (IP-10) [117], the secretion of interleukin 1 receptor antagonist and the synthesis of leukotriene [116], cholesterol acyltransferases-1 and cyclooxygenase 2 [118].

Leptin promotes the inflammatory infiltrates by acting as a monocyte/macrophage chemoattractant [119] and by stimulating phagocytic function via phospholipase activation [99], the expression of adhesion molecules and the secretion of proinflammatory cytokine such as TNF-α (in an early phase), IL-6 (in a tardive phase), and IL-12 in monocytes [120]. Finally, leptin has been found to increase the oxidative stress in macrophages [121]. 

Leptin may also promote the survival and maturation of DCs via the PI3K-Akt signaling pathway, and functionally direct these cells toward Th1 priming increasing their production of proinflammatory cytokines [122].

Leptin seems to behave as a prosurvival cytokine also for neutrophils, delaying the mitochondrial release of cytochrome C and second mitochondria-derived activator of caspase, and the activation of caspase-3 and -8 in these cells [115]. Leptin promotes the expression of CD11b, neutrophils chemotaxis [113], and secretion of oxygen radicals through direct and indirect mechanisms [115].

In addition, leptin enhances survival, chemotaxis, cytokine release and migration in eosinophils and basophils, as well as the expression of adhesion molecules, such as ICAM-1 and CD18 [99]. Leptin and LepR expression has been reported in mast cells suggesting paracrine and/or autocrine pro-inflammatory effects on mast cells [123] as confirmed by the opposite effects of leptin deficiency [124].

Leptin has been demonstrated also to influence the adaptive immune response.

Leptin modulates the activation and proliferation of human T lymphocytes [125], plays a role in maintaining lymphocyte survival by inhibition of Fas-mediated apoptosis. It polarizes T cells toward a Th1 response inducing the synthesis of IL-2, IL-12, and IFN-γ and inhibiting the production of IL-10 and IL-4 [125]. Contrariwise, leptin deficiency in mice and humans leads to a shift from Th1 to Th2 phenotype and to a reduction in total CD4+ T cell [112,126]. Accordingly, LepR is much more expressed in peripheral CD4+ T cells than in CD8+ T cells [99]. Leptin facilitates Th17 cells, which are a CD4+ proinflammatory T-cell subset generated to hinder infections, such that LepR on T-cell membrane is required for full Th17 differentiation [127]. Leptin is also secreted by Th1/Th17 lymphocytes potentiating its own effect by an autocrine loop of secretion [128]. Conversely, leptin inhibits the expansion of human regulatory Foxp3+ CD4+ CD25+ T cells (Treg) [129] which are mediators of immune tolerance and limit inflammation.

Finally, although fewer studies addressed this issue, leptin seems to modulate B-cell compartment, activating B cells to secrete inflammatory cytokines (i.e., TNF-α, IL-6, IL-10) [130].

Immune system impairments attributed to leptin deficiency, as in cases of genetic mutations, are treatable with leptin replacement therapy. Indeed, in several prospective case studies, leptin replacement therapy (with subcutaneous human recombinant methionyl leptin) for 4 to 8 months has been shown to improve immune dysfunction in patients with generalized forms of lipodystrophy, normalizing absolute number and relative percentages of T lymphocyte subsets and increasing TNF-α secretion in PBMC [131]. In addition, long-term (up to four years) leptin replacement therapy in congenital leptin deficiency caused lymphocytes, neutrophils, and monocytes increase and restored Th1/Th2 cytokine balance [132] along with T-cell responsiveness [133].

The immunostimulatory potential of leptin cannot be neglected in vaccines development either [134]. Researchers explored the adjuvant role of leptin in mucosal (either intragastric or intranasal) vaccination against a Gram-positive bacterial pneumonia caused by *Rhodococcus equi* infection [135]. In this study, only mice vaccinated with LL-VapA (a native *Lactococcus lactis* vector expressing virulence-associated protein-A of *Rhodococcus equi*) combined with LL-leptin (a recombinant strain of *Lactococcus lactis* secreting biologically active leptin) were able to develop protective immunity [135]. In detail, the co-administration of LL-Lep strain, enhanced the Th1 response evoked by intranasally LL-VapA-immunized mice whereas only the co-administration of LL-Lep induced a protective immune response in intragastric vaccinated mice, associated with a mixed Th1/Th2 response [135]. Similarly, LepR knockout mice (db/db) were not protected by prophylactic vaccination against *Helicobacter pylori* [136], indicating the crucial role of leptin and its signaling in the generation of host protective immune response.

## 9. Leptin In Viral Infections

The role of leptin in the immune system response to infections has been investigated mostly working on mice models. Many researchers used leptin knockout (*ob/ob*) and *db/db* mice. However, in both *ob/ob* and *db/db* models the lack of leptin-driven satiety cues results in hyperphagia and obesity, which complicates data interpretation. Thus far, it has been difficult to differentiate the effects of energy imbalances from the effects that leptin directly exerts on immune function, as they are experimentally, as well as physiologically, interconnected. 

For instance, thymic atrophy, increased circulating monocytes, and reduced lymphocytes number, NK cell cytotoxicity, and antigen-specific T-cell proliferation have been reported in *ob/ob* and/or *db/db* mice [112].

During influenza A pneumonia infection, *db/db* or malnourished mice showed reduced viral clearance, lung IFNγ level and survival [137]. However, in mice lacking functional LepR uniquely in macrophages and lung epithelial cells, mortality rate was reduced, suggesting that leptin signaling in non-myeloid cells, such as NK and T cells, mostly mediates the immunomodulatory role of leptin upon virus challenge [138]. Both diet- and genetic-induced obese mice exhibited greater lung damage during the pH1N1 infection [67]. Although a number of potential mechanisms may be responsible, Tregs are critical regulators of immunopathology and have been shown to limit the inflammatory response to respiratory syncytial and influenza viruses in mice [139,140]. Tregs isolated from lung airways of DIO mice during pH1N1 challenge are fewer and with impaired suppressive capacity compared to Tregs from lean mice [141]. The key role of leptin in the negative control of Treg has been confirmed by the opposite observation of an increased percentage of peripheral Tregs in *ob/ob* mice compared to wild-type mice [142]. In human bronchial epithelial cells infected with respiratory syncytial virus, leptin was overexpressed enhancing the differentiation of Th17 subset and ERK1/2 phosphorylation, and suppressing Th2 subset differentiation [143].

In antiretroviral-naïve HIV-1-infected patients, serum leptin levels were inversely associated with viral replication, independent of adipose tissue amount and disease progression (i.e., cellular activation and innate immunity effector levels) [144]. In HIV infection, leptin levels correlates with CD4+ T lymphocytes number and increases along with them during highly active antiretroviral therapy [145]. Interestingly, leptin stimulation of HIV+ monocytes, which are characterized by an increased expression of LepR [145], partially inhibited the production of reactive oxygen species (ROS), an indicator of programmed cell-death in these cells, resulting in an attenuation of HIV-induced oxidative burst [146]. This is in contrast with the expected proinflammatory function of leptin and could be explained either by the induction of a hypo-inflammatory/anergy state in HIV+ monocytes, as observed in other inflammatory conditions such as sepsis under LPS stimulation, or by an anti-apoptotic effect of leptin on these cells [116].

Encephalomyocarditis virus infection in *ob/ob* mice caused a more severe inflammatory myocardial damage through enhanced expression of TNF-α compared to that of wild-type mice [147]. Similarly, *db/db* mice exhibited higher susceptibility to Coxsackie virus B4 infection than heterozygous normal (*db*/+) and normal (+/+) genotypic mice [148].

Many viruses (i.e., influenza, hepatitis B, HIV, Epstein Barr, Sars-Cov2) are capable of inducing SOCS3 to enhance self-replication and evade host immunity. Indeed, SOCS3 negatively regulates signaling leptin as well as of various cytokines and growth factors (i.e., IFN, IL-6, and G-CSF) by inhibiting IFN-α/β JAK/STAT pathways [149]. Therefore, an upregulation of SOCS3 during viral infections may, both directly and by decreasing leptin expression, dampen innate and adaptive immunity. 

Notably, although lymphopenia and the predominance of innate immune macrophages which are features of the SARS-CoV2 infection could reflect a strategy adopted by the CoV to suppress host antiviral response, the finding that reduced lymphocyte counts correlated with increased leptin levels may suggest that leptin-induced monocyte changes may also result in insufficient virus-specific T-cell priming [150].

Furthermore, influenza A virus as well as other viral infections produce endoplasmic reticulum (ER) stress which in turn activates unfolded protein response (UPR) pathways (i.e., inositol-requiring protein-1). The activation of UPR has been recognized as critical for influenza A viral replication because it impairs T- and B-cell development, plasma cells differentiation and leptin signaling [151].

## 10. Leptin in Obesity: What Role during Viral Infections?

Because leptin reduces food intake and body weight, the coexistence of high serum leptin concentrations and obesity is widely interpreted as evidence of an attenuation of leptin signaling termed “leptin resistance” [152]. However, there are currently no methods for assessing leptin sensitivity in a clinical setting. Although hyperleptinemia is often considered as a key marker of leptin resistance, it should be noted that serum leptin concentrations depend not only on its transcription levels but also on its clearance rate and the efficiency of its signal transduction [153]. Moreover, the observation that obese individuals exhibit changes in appetite and energy expenditure after moderate weight loss and that these changes are blunted by the administration of leptin, suggests that the high leptin concentrations in obesity may be biologically relevant [154]. Accordingly, in DIO mouse model challenged with H1N1 influenza infection, hyperleptinemia was associated with increased mortality, viral spread, and lung inflammation, which were all significantly improved by the administration of anti-leptin antibody [155].

In this complex contest in which it is not feasible to define leptin resistance in a universal and quantifiable manner, several mechanisms that may underlie the attenuated responsiveness to leptin in vivo have been described. First, leptin signaling is potently antagonized by an upregulation of SOCS3 which specifically binds to LepR via phosphorylated tyrosine-985, resulting in JAK2 dephosphorylation, so that the key pathway affected is JAK/STAT [149]. Leptin itself, when chronically elevated as in the obese state, can induce SOCS3 expression as a mechanism of negative feedback. There are also multiple protein tyrosine phosphatases (i.e., RPTPe, PTP1B and TCPTP) that are capable of dephosphorylating JAK2 and that are upregulated in a high-fat diet and obesity [156]. Second, ER stress triggered by chronic positive energy balance activates the UPR that, if ER stress persists, switches from a pro-survival to a pro-apoptotic response and induces leptin resistance by inhibiting leptin-induced STAT3 phosphorylation [157]. An additional factor implicated in leptin resistance pathogenesis is the downregulation of the short- and long-form isotypes of LepR [152]. Leptin transport across the blood-brain barrier occurs via short-form leptin receptors, whose downregulation in the hypothalamus, in combination with high serum leptin concentrations, leads to a saturable, unidirectional transport system [152]. Finally, circulating factors such as C-reactive protein and clusterin may bind leptin altering its bioavailability and activity [158].

**Figure 3:**
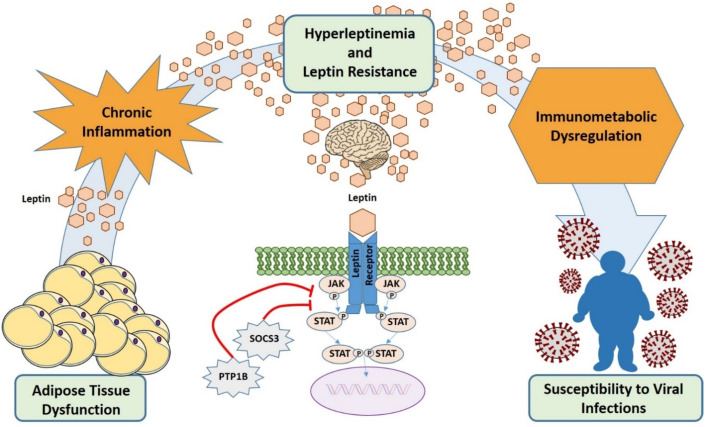
Hyperleptinemia and leptin resistance contribute to immune dysregulation and increased susceptibility to viral infections in obesity. In obesity, adipose tissue produces high levels of leptin (hyperleptinemia) that causes desensitization of target cells for leptin signaling (leptin resistance) by several mechanisms including overexpression of SOCS3. This results in diminished immune cells response and, finally, in an increased susceptibility to infections. In addition, the hyperleptinemia contributes to the obesity low-grade inflammatory background.

Leptin resistance has been demonstrated in immune cells such as NK cells [159], T cells [160], and monocytes [161], and this might contribute to suboptimal immune responses to viral infection in obese individuals. LepR desensitization could be perceived by immune cells as a condition of leptin deficiency, leading to immune dysfunction similarly to malnutrition and genetic leptin deficiency (Figure 3).

Nevertheless, both direct and indirect effects of leptin on the immune system are likely to account for the immune defects observed in leptin-resistance conditions. In fact, when *db/db* bone marrow cells were transplanted into wild-type mice to generate bone marrow chimeras, thymus cellularity and cellular and humoral immune responses resulted normal, suggesting that direct effects of leptin on bone marrow-derived cells and thymic stromal cells are not necessary for T-lymphocyte maturation and immune response. Rather, the major effects of leptin on immune system are indirect via changes in the systemic environment [162].

After all, *ob/ob* and *db/db* mice display multiple neuroendocrine and metabolic alterations. These include the overactivation of the hypothalamic-pituitary-adrenal axis with subsequent hypercortisolism, decreased activity of the sympathetic nervous system, and altered production of various neuropeptides, which are all known to have immunomodulatory effects. In line with this, the negative effects of leptin deficiency on the hepatic innate immune system appear mostly related to the decreased sympathetic tone and β-adrenergic signaling [163].

Instead, with regard to hypercortisolism, even though it is known to induce immune suppression and favor thymic atrophy, in *db/db* mice the relative proportions of CD4+, CD8+ and CD4 and CD8 double positive thymocyte populations are not altered, in contrast with the preferential depletion of CD4 and CD8 double positive thymocytes induced by corticosteroid excess [164], suggesting for leptin a noncorticosteroid-related mechanism of action.

In obesity, adipose tissue dysfunction leads to chronic inflammation not only locally [165] but also in the brain. In particular, hypothalamic inflammation observed in chronic inflammatory conditions such as obesity or upon fat rich diet [166] is considered crucial in leptin resistance development.

In the hypothalamus, leptin activates the sympathetic nervous system [101] and, to a lesser extent, inhibits the hypothalamic-pituitary-adrenal axys [102]. In obesity, as result of leptin resistance at central level, the sympathetic nervous system, which has a major anti-inflammatory role in the brain-immune cross-talk, results less activated [101], thus fostering obesity low-grade inflammatory background [99].

Therefore, if excess leptin secretion from adipocytes can be envisaged as an inflammatory signal that induces proinflammatory environmental and cellular changes, on the other direction chronic inflammation due to metabolic, infectious, or autoimmune diseases impairs leptin signaling as a part of a maladaptive response.

Said that cytokine storms play an important role in the process of COVID-19 aggravation and are considered one of the major determinants of acute respiratory distress syndrome and multiple-organ failure, leptin levels have been found to be not only increased in COVID-19 patients compared with healthy controls, but also significantly higher in severe COVID-19 patients than in mild patients [150]. Notably, in this cohort leptin correlated with BMI, decreased lymphocyte counts and disease progression, showing high consistency with TNF-α and CXCL-10 in predicting disease severity. Leptin correlated with monocyte M1 (inflammatory) polarization in COVID-19 patients and mechanistic experiments revealed that leptin activated STAT3/NF-*κ*B signaling and promoted M1-polarization marker gene and TLR gene expression in immortalized monocyte THP-1 cells [150]. Therefore, it seems that, upon infection, subjects with fat mass excess are prone to produce more leptin which in turn activates monocytes promoting a positive feedback loop and severe cytokine storms.

Leptin can also be inhibited by ACE2 via alamandine production and activation of MrgD-receptor/c/Src/p38MAPK pathway [167]. Therefore, it has been hypothesized that in obese patients infected by SARS-CoV-2, the impairment of ACE2 function due to viral binding may further increase the leptin levels. Consequently, hyperleptinemia combined with local ACE2-angiotensin II dysbalance could contribute to the hyperinflammatory pulmonary response found in obese patients [168].

While impairments attributed to leptin genetic mutations are treatable with recombinant methionyl human leptin administration, as result of leptin resistance replacement therapy in individuals with obesity does not provide therapeutic benefit.

However, in leptin resistance conditions in which vaccine-induced immune responses are dampened, the use of SOCS3 and protein tyrosine phosphatase-1B (PTP1B) inhibitors as viral vaccine adjuvants has been proposed since it may help restore immune homeostasis [134].

Another potential therapy candidate to prevent obesity-related vaccine failure is represented by chemical chaperones that, by decreasing ER stress induced by obesity, can improve leptin sensitivity [169]. Also saponins derived from fungal endophytes could be potential inhibitors of leptin, reverse its resistance in obesity, and potentiate host immune response against multiple diseases [170].

## 11. Conclusions

Metabolic complications, nutritional aspects, physical inactivity, and the chronic imbalance in the hormonal and adipocytokine microenvironment are major determinants in the severity of viral infections in obesity.

Besides the pleiotropic mechanisms by which obesity impairs immunity, the higher leptin concentrations which characterize obesity substantially contribute to such dysregulation of the immune response.

Indeed, chronic inflammatory states due to metabolic (i.e., obesity) or infectious diseases may increase leptin concentrations and lead to leptin resistance further fueling inflammation.

On the other hand, leptin plays an important role in numerous metabolic and immunologic functions, and impairments of leptin signaling due to leptin resistance appear to hamper these processes and possibly contribute to impaired vaccine-induced immune responses. 

However, many aspects concerning leptin interactions with inflammation and immune system, as well as the therapeutical approaches to overcome leptin resistance and reduced vaccine effectiveness in obesity remain a challenge for future research.

## Data Availability

No new data were created or analyzed in this study. Data sharing is not applicable to this article.
